# The fallopian tube as origin of ovarian cancer: Change of diagnostic and preventive strategies

**DOI:** 10.1002/cam4.2725

**Published:** 2019-11-25

**Authors:** Satoru Kyo, Noriyoshi Ishikawa, Kohei Nakamura, Kentaro Nakayama

**Affiliations:** ^1^ Department of Obstetrics and Gynecology Shimane University Faculty of Medicine Izumo Shimane Japan; ^2^ Department of Pathology Shimane University Faculty of Medicine Izumo Shimane Japan

**Keywords:** BRCA mutations, cancer biology, cancer genetics, gynecological oncology

## Abstract

Ovarian cancer is the leading cause of gynecologic cancer death in the world, and its prevention and early diagnosis remain the key to its treatment, especially for high‐grade serous carcinoma (HGSC). Accumulating epidemiological and molecular evidence has shown that HGSC originates from fallopian tube secretory cells through serous tubal intraepithelial carcinoma. Comprehensive molecular analyses and mouse studies have uncovered the key driver events for serous carcinogenesis, providing novel molecular targets. Risk‐reducing bilateral salpingo‐oophorectomy (RRSO) has been proposed to reduce the subsequent occurrence of serous carcinoma in high‐risk patients with *BRCA* mutations. However, there is no management strategy for isolated precursors detected at RRSO, and the role of subsequent surgery or chemotherapy in preventing serous carcinoma remains unclear. Surgical menopause due to RRSO provides a variety of problems related to patients’ quality of life, and the risks and benefits of hormone replacement are under investigation, especially for women without a previous history of breast cancer. An additional surgical option, salpingectomy with delayed oophorectomy, has been proposed to prevent surgical menopause. The number of opportunistic salpingectomies at the time of surgery for benign disease to prevent the future occurrence of HGSC has increased worldwide. Thus, the changing concept of the origin of serous carcinoma has provided us a great opportunity to develop novel diagnostic and therapeutic approaches.

## Graphical abstract

Accumulating epidemiological and molecular evidence has shown that high‐grade serous carcinoma originates from fallopian tube secretory cells through serous tubal intraepithelial carcinoma. The changing concept of the origin of serous carcinoma has provided us a great opportunity to develop novel diagnostic and preventive approaches.

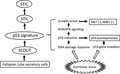

## 1 INTRODUCTION

In the United States of America and Japan, ovarian cancer accounts for 2.5% and 3.1% of cancer diagnoses and is the fouth and ninth leading cause of cancer‐related death, respectively.^1^, ^2^ While the cure rate of patients with disease confined to the ovary is over 90%,^3^, ^4^ those with disseminated or metastatic lesions have 5‐year survival rates of 25%‐30%.^5^, ^6^, ^7^ The prognosis of high‐grade serous carcinoma (HGSC) is particularly poor among the histological types, and prevention and early detection of this subtype are urgently needed. Significant efforts have been made to diagnose ovarian cancer earlier in the course of cancer development, classically using transvaginal ultrasonography with CA‐125 as a serum marker, but there is no definitive screening approach that reduces ovarian cancer mortality.^8^ This review summarizes a recent revolutionary change in the concept of the site of origin of HGSC that affects current ovarian cancer prevention strategies.

## 2 HISTORICAL ASPECTS OF THE FALLOPIAN TUBE AS A POTENTIAL ORIGIN OF SEROUS CARCINOMA

Ovarian cancer had been believed to arise from ovarian surface epithelial cells. However, this concept has changed revolutionarily in HGSC, a major histological subtype of ovarian cancer, in the past two decades. It all began with the discovery of the *BRCA1* and *BRCA2* tumor suppressor genes. Approximately 5%‐10% of ovarian cancers are attributed to inherited germline mutations of susceptible genes, and about 90% of such cases involve mutations of *BRCA1* or *BRCA2* genes.^9^, ^10^, ^11^ Mutation carriers have an increased risk of ovarian cancer, at 40%‐60% at the age of 70 years,^12^ while a lifetime risk of general population to develo Oral contraceptive (OC) is 1 in 75 (1.3%).^13^ Risk‐reducing bilateral salpingo‐oophorectomy (RRSO) has therefore been recommended for women with hereditary ovarian cancer syndrome at age 35‐40 years for *BRCA1* mutation carriers and at age 40‐45 years for *BRCA2* carriers.^14^, ^15^, ^16^

At the beginning of 2000, there were several reports of epithelial abnormalities of the fallopian tubes in RRSO specimens, called serous tubal intraepithelial carcinoma (STIC).^17^, ^18^, ^19^, ^20^ In 2005, the Sectioning and Extensively Examining the FIMbriated end of the fallopian tube (SEE‐FIM) protocol was introduced by the Brigham and Women's Hospital group for routine analysis of fallopian tubes of women with *BRCA* mutations or with a family history of breast and/or ovarian cancer.^21^ This protocol triggered increased reports of STIC or early serous carcinoma; approximately 2% of the cases with RRSO had such early lesions, mainly in the fimbriae of the fallopian tubes.^22^, ^23^ Detecting precancerous or early serous carcinoma in the fallopian tubes of *BRCA* mutation carriers led to the hypothesis that serous carcinoma in the ovary or other pelvic sites originates from the fallopian tubes. Supporting this hypothesis, approximately 50% of patients with HGSC were found to have co‐existing STIC when the SEE‐FIM protocol was applied.^24^ Subsequent studies reported a varied frequency (20%‐60%) of this association, but this inconsistency may be due to the difficulty of identifying intact fallopian tubes likely being involved in ovarian masses.^25^ The most striking findings for the linkage of STIC and serous carcinoma are common somatic mutations in *TP53*,^24^, ^26^, ^27^ as well as other molecular markers, such as elevated stathmin 1,^28^ shortened telomeres,^29^ and cyclin E amplification,^30^ shared with these lesions.

## 3 BROAD SPECTRUM OF TUBAL PRECURSOR LESIONS

**Figure 1:**
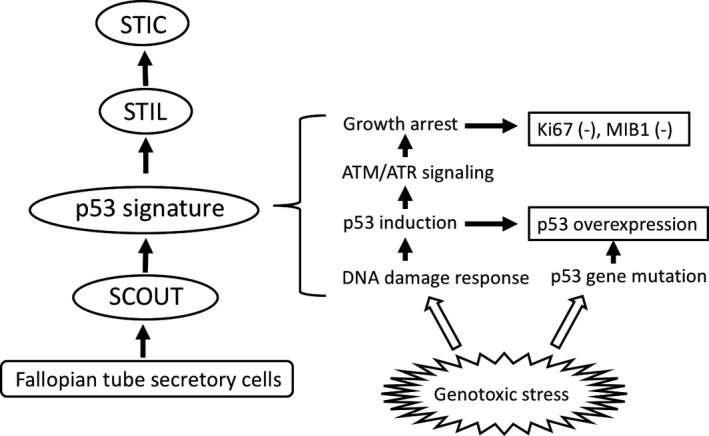
Concept of the tubal precursors. Some secretory cells in the fimbria exhibit expanded growth, leading to secretory cell outgrowth (SCOUT), and are likely to undergo genotoxic oxidative stress, probably due to exposure to follicular fluid at the time of ovulation. The DNA damage response will then be induced by activating the p53 pathway, leading to subsequent activation of ATM/ATR signaling and cell cycle arrest. Thus, p53 signature is characterized as negative for Ki‐67 or MIB1 staining. Genotoxic stress or some other factors in p53 signature cells induces or causes *p53* gene mutation, leading to the development of serous tubal intraepithelial lesions (STILs) and serous tubal intraepithelial carcinoma (STIC)

STIC and serous carcinoma have frequent *p53* mutations and, therefore, exhibit p53 overexpression on immunohistochemistry. However, it was reported that small segments of strongly p53‐positive cells were commonly observed in fimbriae, irrespective of *BRCA* status, called the “p53 signature”.^26^ Careful histochemical analysis showed that the p53 signature is predominant in the fimbriated end, especially in nonciliated (secretory) cells, and p53 signature is more frequently present in association with STIC.^26^ The p53 signature was frequently associated with γ‐H2AX staining, histochemical evidence of double‐strand DNA breakage,^26^ indicating that it is initiated by DNA damage. Thus, the p53 signature might be a reactive change in response to genotoxic circumstances, such as by exposure to oxidants in follicular fluid in the post ovulation period. Approximately 50% of p53 signatures are known to have gene mutations in *p53*, similar or identical to those observed in STIC.^26^ Thus, such genotoxic circumstances in fimbriae may induce not only up‐regulation but genetic mutations in *p53*, the latter of which may cause progression to STIC (Figure 1). Interestingly, patients of Li Fraumeni syndrome with *p53* germ‐line mutations showed markedly increased frequency of *p53* signature in the distal fallopian tube,^31^ indicating the pivotal role of *p53* gene mutation in establishing p53 signature.

**Figure 2:**
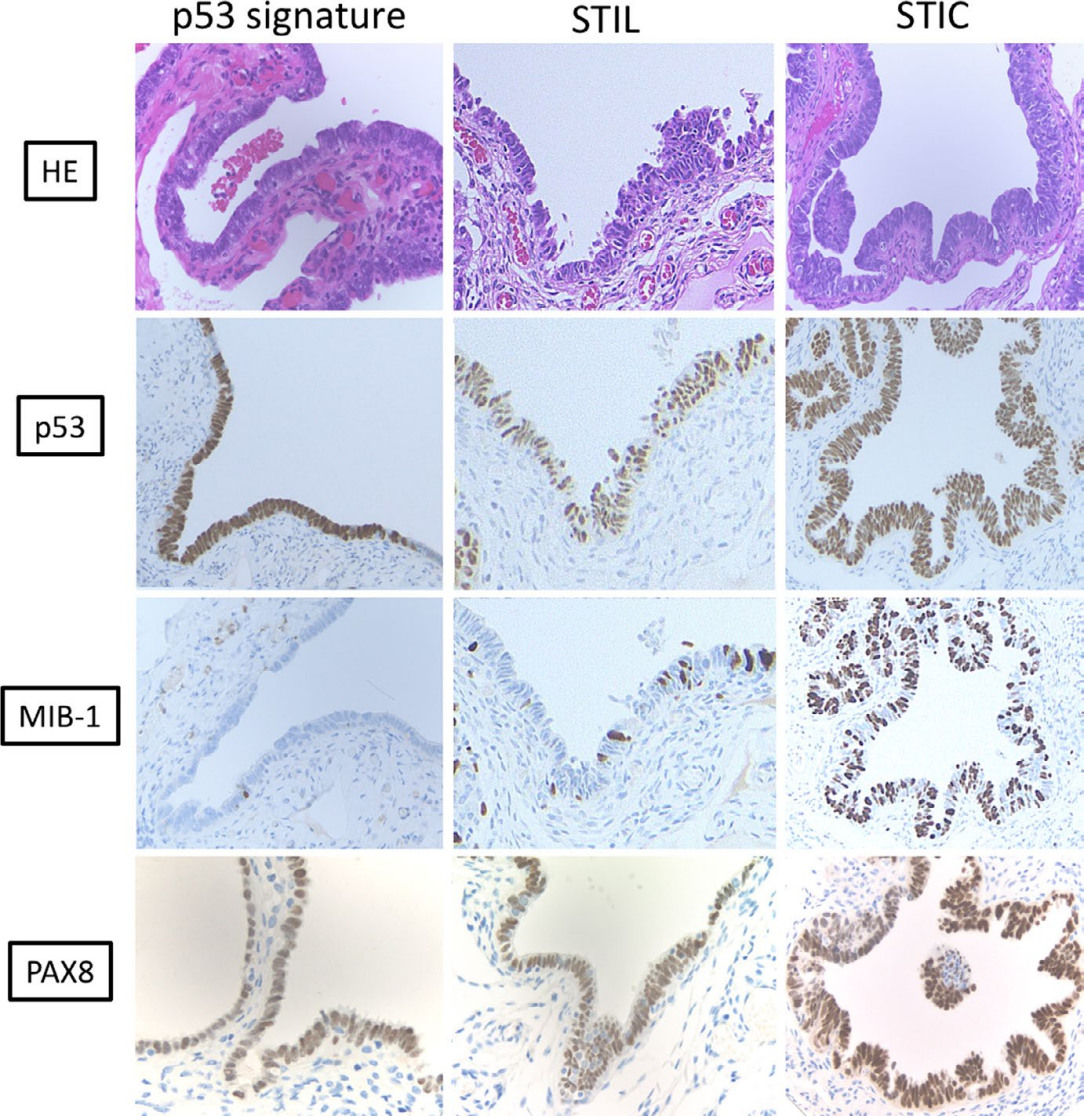
Representative pathological findings of precursors to high‐grade serous carcinoma. The p53 signature exhibited normal morphology with p53 overexpression, but without high proliferative activity, lacking MIB1 expression. Serous tubal intraepithelial carcinoma (STIC) shows cytological atypia and loss of polarity, p53 overexpression due to *p53* mutation, and high proliferative activity with significant MIB1 expression. Serous tubal intraepithelial lesions (STILs) have intermediate findings of morphology and proliferative activity between the p53 signature and STIC, which are considered transitional lesions. All of these precursors are composed of PAX8‐positive secretory cells

In two types of tubal epithelial cells, so‐called secretory and ciliated cells, the former are less matured and thought to be vulnerable to transformation, and, in fact, secretory cells were proven to be the most susceptible to DNA damage in vitro.^32^ The proliferative activity of the p53 signature has been demonstrated to be low (Figure 2).^26^ This is consistent with the fact that DNA damage facilitates ATM/ATR‐regulated signaling pathways that result in cell cycle arrest.^26^ However, once the p53 signature progresses to STIC, it acquires high‐growth proliferative activity with high Ki‐67 or MIB1 expression and cytologic atypia, as well as loss of cellular polarity^26^, ^33^ (Figure 2). Of particular interest is that transitional lesions between the p53 signature and STIC are often found, with intermediate proliferative and morphological characteristics, called serous intraepithelial lesions (STILs).^33^, ^34^ The presence of these transitional lesions may further suggest that the p53 signature is a precursor of STIC.

A candidate precursor of the p53 signature has also been proposed, named secretory cell outgrowth (SCOUT), and is usually located at more proximal sites of the tube than the p53 signature. SCOUT is defined as consisting of a row of at least 30 secretory epithelial cells with a pseudostratified benign appearance and low proliferative activity that is not interrupted by ciliated differentiation.^35^ SCOUT does not usually display alterations in p53, either by immunostaining or p53 sequence analysis. Continuity of SCOUT, p53 signature, and serous carcinoma can, on occasion, be demonstrated and shown to share identical *p53* mutations. Thus, SCOUT may be a potential precursor of the p53 signature, and the p53 signature may be a subset of SCOUTs.

Several genetic studies have been performed to clarify the molecular relationship between tubal precursor lesions and HGSC. The initial laser‐captured microdissection analysis followed by target sequencing of *p53* indicated that 57% of p53 signatures had *p53* gene mutations, most of which were missense mutations, while all STICs and all STIC/ovarian cancer pairs shared identical *p53* mutations.^26^ Subsequent studies, however, showed that *p53* mutations were detected in all HGSCs analyzed, and the identical mutations were detected in most, but not all, STICs and concurrent HGSCs.^27^ Recent comprehensive genomic analysis by next‐generation sequencing provided striking evidence that the p53 signature or STIC had the ancestral clone for the observed cancers.^36^ The majority of tumor‐specific alterations in ovarian cancers were commonly present in STICs, including those affecting *TP53, BRCA1*, *BRCA2,* or *PTEN*. Ovarian cancers contained additional genetic changes, indicating that tubal precursor lesions represented daughter clones of the ovarian cancers. Evolutionary analyses reveal that p53 signatures and STICs are precursors of ovarian carcinoma and identify a window of 7 years between development of a STIC and initiation of ovarian carcinoma, with metastases following rapidly thereafter.

## 4 IDENTIFICATION OF DRIVER MUTATIONS FOR SEROUS CARCINOGENESIS

To clarify the molecular mechanisms through which serous carcinoma develops from the fallopian tubes, comprehensive genomic analyses of HGSC have been performed, as the Cancer Genome Atlas (TCGA).^37^ This analysis found that *TP53* mutation was highly prevalent (96%), as expected, and additional genes including *NF1*, *BRCA1*, *BRCA2, RB1*, and *CDK12* also had somatic mutations with relatively low but statistically significant frequencies. Somatic DNA copy number aberrations were identified, including CCNe1, MYC, and MECOM, each highly amplified in >20% of tumors. The pathway analyses showed that RB‐related (67%) and RAS/PI3K‐related (45%) signaling pathways were activated, and homologous recombination defects (HRDs) were observed in up to 49% of cases, via *BRCA1* promoter hypermethylation and silencing, as well as its germline and somatic mutations. These findings indicate that the mutational spectrum marks HGSC as completely distinct from other histological subtypes of ovarian cancer, with extremely high *TP53* mutations and frequent HRDs.

Mouse models have been established to uncover the potential origin and the molecular pathways for serous carcinogenesis. Conditional knockout of *BRCA*, *TP53,* and *PTEN* with the Cre‐loxP system in fallopian tube secretory cells (FTSECs) was performed^32^ (Table 1). *BRCA1* ^mut^, *TP53* ^mut^, *PTEN^−/−^* and *BRCA2* ^mut^, *TP53* ^mut^, *PTEN^−/−^* mice developed STIC and HGSC. The *TP53^−/−^, PTEN^−/−^* mice did not progress past the pre‐invasive stage of the disease, suggesting that *BRCA* alterations are necessary for the progression of HGSC *BRCA2^−/−^, TP53* ^mut^ mice without *PTEN* alterations developed STIC, but none showed invasive tumors, the disease latency was much longer, and tumorigenesis was inefficient, suggesting that *PTEN* alterations are required for tumor initiation and progression, cooperating with *BRCA* and *TP53* deletion/mutation. Consistent with this, a recent study demonstrated that PTEN expression was markedly reduced or absent in one‐third of human STICs.^38^

**Table 1:** Identification of driver gene mutations required for serous carcinogenesis in mouse model or in vitro carcinogenesis model

Genotype	Knockout mouse model (Ref. 32)			
	Number of mice	STIC	Ovarian metastasis	Peritoneal metastasis
BRCA1*^−/−^*, TP53MT, PTEN*^−/−^*	4	4/4 (100%)	1/4 (25%)	1/4 (25%)
BRCA1^+^ *^/−^*, TP53MT, PTEN*^−/−^*	12	10/12 (83%)	6/12 (50%)	8/12 (67%)
BRCA2*^−/−^*, TP53MT, PTEN*^−/−^*	12	9/12 (75%)	9/12 (75%)	8/12 (67%)
BRCA2^+^ *^/−^*, TP53MT, PTEN*^−/−^*	3	3/3 (100%)	3/3 (100%)	2/3 (67%)
TP53*^−/−^*, PTEN*^−/−^*	6	4/6 (67%)	0/6 (0%)	0/6 (0%)
BRCA2*^−/−^*, TP53*^−/−^*	11	11/11 (100%)	NR	3/11 (27%)

Trangene	Transgenic mouse model (Ref. 39)			
	Number of mice	p53 signature	STIC	Invasive adenocarcinoma
TAg	34	34/34 (100%)	34/34 (100%)	19/34 (56%)

Introduced genetic factors	In vitro carcinogenesis model (Ref. 40)			
	Number of mice	Conlony formaiton on soft agar	Tumor formation (subcutaneous)	Tumor formation (intraperitoneal)
DN‐p53	4	0	0	0
DN‐p53, KRAS MT	4	0	0	0
DN‐p53, c‐Myc	4	0	0	0
DN‐p53, CA‐AKT	4	+	0	0
DN‐p53, KRAS MT c‐Myc	4	+	4	4
DN‐p53, KRAS MT, CA‐AKT	4	+	4	4

Abbreviations: CA‐AKT, constitutively activated AKT; DN‐p53, dominant negative form of p53; MT, mutation; STIC, serous tubal intraepithelial carcinoma; TAg, SV40 T antigen.

Sherman‐Baust et al established a transgenic mouse model of serous carcinoma, in which SV40 large T‐antigen (TAg) was used as a transgene under the control of mouse mullerian‐specific Ovgp‐1 promoter.^39^ Histological analysis of the fallopian tubes of this mouse showed a variety of neoplastic lesions analogous to those described as precursors (Table 1). Furthermore, invasive ovarian serous carcinoma was observed in 56% of the mice. The TAg cassette in this model is known to inactivate both p53 and Rb. Since *p53* mutation, as well as the inactive Rb pathway, has already been known to be essential for the development of human HGSC by TCGA study, this mouse model seems to be partly consistent with human serous carcinogenesis. However, inactivation of p53 by TAg is not equivalent to *p53* mutation as some *p53* mutations function as dominant negative fashion. Therefore, this model is not completely consistent with human serous carcinogenesis. Furthermore, TAg is likely to generate a variety of chromosomal aberrations and the minimal genetic requirement for carcinogenesis remains unclear in this model.

We recently established an in vitro model for serous carcinoma using primary‐cultured human fimbria cells.^40^ Epithelial cells were isolated from surgically removed fimbriae in patients with benign uterine diseases, subjected to primary culture, followed by immortalization by lentiviral overexpression of *cyclinD*1, *cdk4*, and *hTERT*. The immortal cells exhibited epithelial morphology in vitro and expressed secretory cell markers, indicating that these cells originated from FTSECs, not ciliated cells. Based on TCGA data of human HGSCs, we considered that *TP53* mutation is indispensable for carcinogenesis. To mimic *TP53* mutation, we introduced a dominant negative form of p53 (DN‐p53) into immortalize FTSECs, but no phenotypic change was observed (Table 1). Oncogenic mutated *KRAS* allele or constitutively activated AKT (CA‐AKT) was then introduced, based on the TCGA data that RAS/MAPK and/or PI3K/AKT pathways are frequently activated in human HGSC. Furthermore, *c‐Myc*, whose gene amplification was frequently observed in human HGSC, was also overexpressed. Overexpressing DN‐p53/mutant *KRAS*/ *c‐Myc* or DN‐p53/mutant *KRAS*/CA‐AKT successfully led to tumorigenic phenotypes in mice (Table 1), and the histology of mouse xenografts was grossly, histologically, and immunohistochemically similar to human HGSCs.

Taken together, these mouse or in vitro carcinogenesis models support the concept that a total of 3 genetic hits are required for the development of HGSC from FTSECs, in which *TP53* mutation is indispensable and the additional 2 hits, including *BRCA* mutation (or HRD), PI3K‐APT, Ras‐MAPK signaling, or c‐Myc amplification (overexpression), confer the serous carcinoma phenotype.

## 5 STRATEGY TO PREVENT OVARIAN CANCER IN HIGH‐RISK POPULATIONS

Oral contraceptives have been known to have a preventive effect on ovarian cancer occurrence in *BRCA1/2* carriers.^41^, ^42^, ^43^ The precise mechanism by which OCs have protective effects remains unclear, but may be at least partly due to inhibition of ovulation that may reduce the opportunity for fimbriae to contact the ovarian surface in every menstrual cycle. RRSO has been established as protective surgery against ovarian cancer in the high‐risk population with *BRCA1* or *BRCA2* mutations.^14^, ^15^, ^16^ A prospective multicenter cohort study with over 2000 high‐risk women with *BRCA1* or *BRCA2* mutations demonstrated that RRSO resulted in decreased ovarian cancer risk (HR 0.15‐0.31), as well as cancer‐specific mortality (HR 0.25), compared with the population without RRSO.^44^ Another multicenter study of RRSO enrolled over 1000 women with *BRCA1* or *BRCA2* mutations who self‐selected RRSO or observation and demonstrated an 85% reduction in *BRCA1*‐associated gynecologic cancer risk (HR 0.15) and a 72% reduction in *BRCA2*‐associated breast cancer risk (HR 0.28). Protection against *BRCA1*‐associated breast cancer or *BRCA2*‐associated gynecologic cancer was observed, but it was not statistically significant,^45^ in which relatively low incidence of *BRCA2*‐associated gynecologic cancers in the cohort (two in the surveillance cohort, zero in the RRSO cohort) limits conclusions regarding the impact of RRSO on the risk of subsequent *BRCA2*‐associated gynecologic cancers.

In contrast to these protective benefits, RRSO is associated with surgical menopause, generating various long‐term health problems, as well as unfavorable symptoms with climacteric disorders, including sexual dysfunction and https://www.sciencedirect.com/topics/medicine-and-dentistry/vasomotor symptoms, making women reluctant to pursue surgery. Studies on quality of life demonstrated the benefit of hormone replacement therapy (HRT) for *BRCA* mutation carriers after RRSO, with fewer endocrine symptoms and better sexual functioning,^46^, ^47^, ^48^, ^49^, ^50^ as well as decreased bone diseases.^51^, ^52^ The breast cancer risk by taking HRT after RRSO is a great concern for *BRCA* mutation carriers who have not yet developed it. Accumulating evidence has shown that breast cancer risk reduction after RRSO is not changed by HRT.^53^, ^54^ The formulation of HRT may affect the risk of breast cancer; estrogen‐replacement therapy may be preferred over progestin‐containing regimens.^54^, ^55^ A definitive conclusion about the risk and benefit of HRT in high‐risk patients will be reached only through well‐designed, long‐term studies.^56^

Based on the accumulating evidence that HGSC may originate from the fallopian tubes, bilateral salpingectomy (without oophorectomy) may offer a reduced risk of ovarian cancer in *BRCA1* or *BRCA2* mutation carriers and greater peace of mind, while enabling women to delay or avoid surgical menopause and maintain fertility. Prophylactic salpingectomy with delayed oophorectomy has therefore been proposed.^57^ A multicenter Danish trial with early salpingectomy and delayed oophorectomy in *BRCA1/2* mutation carriers (TUBA study) started in 2015,^58^ enrolling patients who self‐selected standard RRSO or risk‐reducing bilateral salpingectomy with delayed oophorectomy (BS/DO) at age 40‐45 years for *BRCA1* mutation carriers and at age 45‐50 years for *BRCA* 2 mutation carriers, in which the primary outcome measures were menopause‐related quality of life. The trial is planned to continue until 2025 with over 500 women. Several similar studies are ongoing.^59^, ^60^, ^61^

## 6 HOW TO DEAL WITH ISOLATED STIC DETECTED AT RRSO?

If STIC is detected at RRSO, what should we do? The incidence of STIC in high‐risk patients who undergo RRSO has been reported to be approximately 2%,^14^, ^15^ but this varied from 0.4% to 11.5% in recent reports including 4279 cases of RRSO from 2006 to 2017^21^, ^22^, ^62^, ^63^, ^64^, ^65^, ^66^, ^67^, ^68^, ^69^, ^70^, ^71^, ^72^, ^73^, ^74^, ^75^, ^76^ (Table 2); the diversity may be due to the lack of a common concept of comprehensive sectioning by the SEE‐FIM protocol. The type of surgery performed at or after RRSO varied, including hysterectomy, omentectomy or lymphadenectomy, and staging surgery, and most patients who underwent such procedures had evidence of disease in the resected organs. Approximately 10% of the patients who underwent peritoneal cytology examination had positive cytology. After the diagnosis of isolated STIC, about 20% of the patients received platinum/paclitaxel‐based chemotherapy. Three (4.5%) of 67 patients with follow‐up records showed subsequent primary peritoneal cancer during the follow‐up period. The clinical significance of positive peritoneal cytology at RRSO also remains unclear, and it has not been determined whether positive cytology is associated with a higher risk of subsequent peritoneal cancer.^77^ Furthermore, there is insufficient evidence that surgical staging or postoperative chemotherapy for patients with isolated STIC at RRSO decreases the occurrence of peritoneal cancer. There is thus no established management concept for high‐risk patients with isolated STIC after RRSO. It has been noted that patients with incidental STIC are more likely to develop peritoneal cancer compared to those with benign findings at RRSO.^73^ Therefore, *BRCA* mutation carriers with incidental STIC after RRSO should be carefully followed‐up, at least by more frequent examination with CA‐125 and imaging by ultrasonography/CT.

**Table 2:** Clinico‐pathological findings of isolated STIC detected at RRSO

Reference	Cases of STIC (STIC/RRSO)	Median age (range)	BRCA status (BRCA1 mt/BRCA2 mt)	Cytology at RRSO (negative/positive)	Surgery at or after RRSO	Chemotherapy	Follow up after RRSO in months (range)	Subsequent cancer
Finch et al^62^	1/159 (0.6%)	64	1/0	1/0	TH + OMT (1)	NR (1)	NR	NR (1)
Carcangju et al^63^	3/50 (6%)	53 (48‐61)	3/0	2/0, NR (1)	TH (1)	ND (3)	44 (7‐87)	None (3)
Lamb et al^64^	4/113 (3.5%)	53 (46‐65)	2/2	3/1	staging (1), ND (3)	C/P (2), ND (2)	NR	None (4)
Medeiros et al^21^	3/26 (11.5%)	51 (43‐66)	1/2	2/1	TH (3), Staging (3)	NR (3)	NR	NR (3)
Callahan et al^65^	3/122 (2.5%)	51 (44‐66)	1/2	2/1	TH (3), Node (2)	C/P (3)	NR	None (3)
Shaw et al^66^	15/176 (8.5%)	NR	8/7	NR (15)	NR	NR (15)	NR	NR (15)
Manchanda et al^67^	6/308 (1.9%)	53 (44‐67)	3/1, unknown (2)	6/0	ND (6)	ND (6)	NR	NR (6)
Mingels et al^68^	14/226 (6.2%)	NR	9/5	NR (14)	NR	NR (14)	NR	NR (14)
Powel et al^69^	16/407 (3.9%)	56 (46‐76)	12/4	13/3	Staging (9)	C/P (4), ND (12)	81 (40‐150)	PPC 1, None (15)
Reitsma et al^70^	3/360 (0.8%)	54 (50‐57)	0/2 (+VUS1)	3/0	ND (3)	NR (3)	12 (2‐26)	None (3)
Wethington et al^22^	12/593 (2%)	54 (39‐77)	5/5, unknown (2)	11/1	TH (7), OMT (7), Biopsy (9), Node (6)	ND (12)	28 (16‐44)	None (12)
Conner et al^71^	11/349 (3.2%)	49 (31‐60)	5/1, BRCA1 or 2 (5)	7/NR (4)	Staging (3)	C/P (2), ND (9)	60 (12‐96)	Elevated CA125 and ascites (1), None (10)
Sharman et al^72^	4/966 (0.4%)	54 (28‐58)	2/2	4/0	ND (4)	NR (4)	NR	NR (4)
Zakour et al^73^	9/246 (3.7%)	57 (36‐76)	8/1	9/0	TH (2), NR (7)	ND (9)	79 (25‐138)	PPC (2) None (7)
Poon et al^74^	3/72 (4.2%)	52 (range NR)	2/1	1/1, NR (1)	ND (3)	ND (3)	79 (45‐108)	None (3)
Miller et al^75^	3/70 (4.3%)	NR	3/0	3/0	Peritoneal and Omental biopsy (3)	NR (3)	NR	None (3)
Lee et al^76^	2/36 (5.6%)	51 (range NR)	1/1	NR (2)	NR (2)	NR (2)	NR	NR (2)
Total	112/4279 (2.6%)	54 (31‐77)	66/36 (+VUS1), unknown 9	Positive ratio 8/75 (10.7%)	TH 15/72 (20.5%) OMT 8/72 (11.1%) staging 16/72 (22.2%) node 8/72 (11.1%) biopsy 12/72 (16.7%)	C/P 11/67 (16.4%)	(2‐150)	PPC 3/67 (4.5%)

Abbreviations: C/P, carboplatin‐paclitaxel; mt, mutation; ND, not done; Node, retroperitoneal lymphadenectomy; NR, not recorded; OMT, omentectomy; PPC, primary peritoneal cancer; RRSO, risk‐reducing bilateral salpingo‐oophorectomy; STIC, serous tubal intraepithelial carcinoma; TH, total hystecrtomy; VUS, variant of uncertain significance.

## 7 CAN OPPORTUNISTIC SALPINGECTOMY REDUCE THE RISK OF OVARIAN CANCER?

Based on the accumulating evidence of the tubal origin of ovarian cancer, opportunistic salpingectomy or tubal ligation in the low‐risk population may reduce the incidence of this type of tumor. Two large‐scale studies were performed,^78^, ^79^ showing that opportunistic salpingectomy at the time of surgery for benign disease significantly reduced epithelial ovarian cancer risk (Table 3).

**Table 3:** Large‐scale retrospective studies for ovarian cancer risk reduction by salpingectomy or related operations

	Madsen et al (Ref. 78/)	Falconer et al (Ref. 79)
Design	Retrospective case‐control	Retrospective cohort
Setting	Registry in Danish population	Registry in Swedish population
Population	13 241 ovarian cancer cases	98 026 cases with hysterectomy
	194 689 age‐matched population control	37 348 cases with hysterectomy with BSO
		34 433 cases with salpingectomy
		81 658 cases with tubal sterilization
		5 449 119 unexposed cohort
Comparator	Observation	Unexposed population
Outcome	Incidence of ovarian cancer	Incidence of ovarian cancer
Impact of hysterectomy		HR 0.79 (95% CI, 0.70‐0.88)
Impact of hysterectomy with BSO		HR 0.06 (95% CI, 0.03‐0.12)
Impact of salpingectomy	OR 0.58 (95% CI, 0.36‐0.95)	HR 0.36 ( 95% CI, 0.52‐0.81)
Impact of tubal sterilization	OR 0.87 (95% CI, 0.78‐0.98)	HR 0.69 (95% CI, 0.64‐0.81)

Abbreviations: BSO, bilateral salpingo‐oophorectomuy; HR, hazard ratio; OR, odds ratio.

In the United States of America, 25 180 girls and women who underwent inpatient hysterectomy from 2008 through 2013, representing a national cohort of 2 036 449 girls and women, were investigated.^80^ There was an increase in the uptake of hysterectomy with BS of 371% across the study period. Based on the epidemiological evidence, a worldwide increase in adopting opportunistic salpingectomy has been observed in society guidelines,^81^ and the Japan Society of Obstetrics and Gynecology made a statement recommending opportunistic salpingectomy at the time of hysterectomy for benign diseases.

## 8 CONCLUSIONS

Determining precisely where these tumors initiate will affect strategies for early detection, such as improved methods of diagnostic imaging that focus on the distal fallopian tube, in addition to the ovary. For example, one can imagine that fallopian tubes or fimbriae may be a target of cytology or biopsy with a specialized apparatus in outpatients, possibly as a screening test especially for high‐risk patients or patients with an ovarian mass or ascites suspicious of HGSC. The driver gene mutations for serous carcinogenesis have been identified, and a total of three genetic factors are required for carcinogenesis, providing novel therapeutic targets. RRSO reduces the occurrence of subsequent peritoneal carcinoma, but may partly be replaced by prophylactic salpingectomy with delayed oophorectomy, avoiding surgical menopause. There are unresolved issues about isolated STIC detected at RRSO. No standard management recommendation is proposed for subsequent surgery and/or chemotherapy. The number of opportunistic salpingectomies in low‐risk patients is increasing dramatically with the concept of tubal origin, and this trend will further continue. Overall, the changing concept of the origin of HGSC provided us a great opportunity to uncover serous carcinogenesis, as well as to develop novel diagnostic and preventive approaches.

## CONFLICT OF INTEREST

Neither of the authors has any conflicts of interest to disclose.
